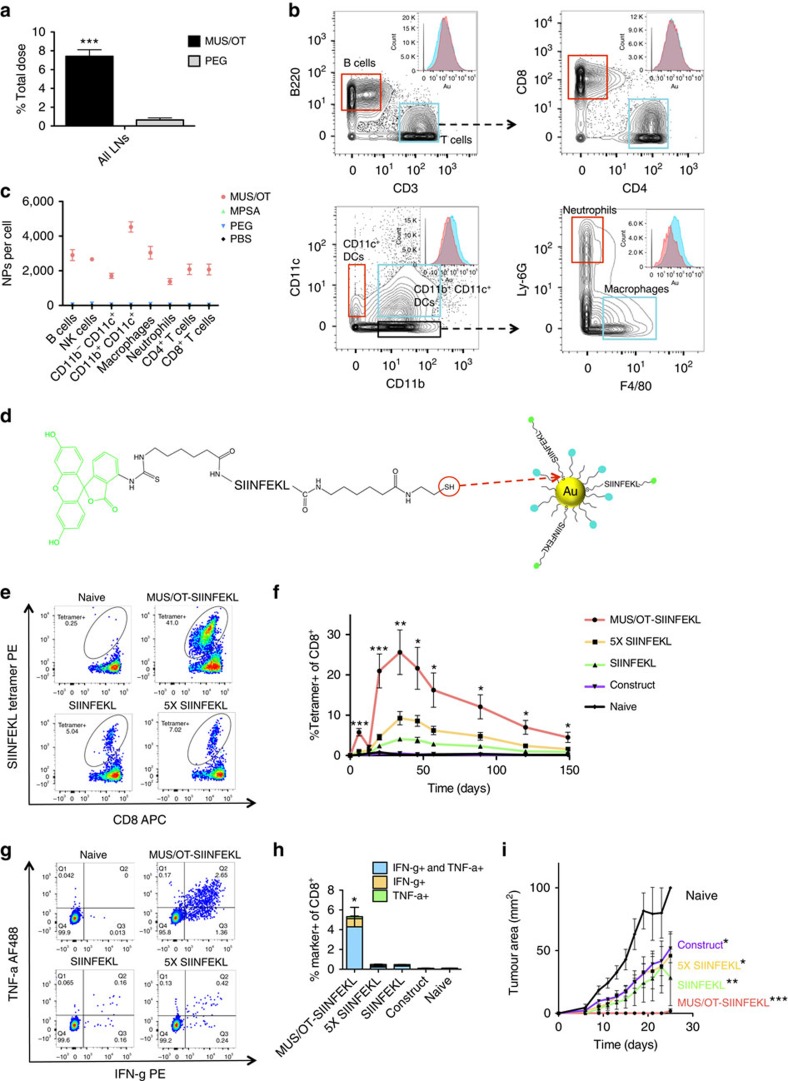# Erratum: High-throughput quantitation of inorganic nanoparticle biodistribution at the single-cell level using mass cytometry

**DOI:** 10.1038/ncomms15343

**Published:** 2017-08-14

**Authors:** Yu-Sang Sabrina Yang, Prabhani U Atukorale, Kelly D. Moynihan, Ahmet Bekdemir, Kavya Rakhra, Li Tang, Francesco Stellacci, Darrell J Irvine

Nature Communications 8 Article number: 14069 ; DOI: 10.1038/ncomms14069 (2017); Published 01 17 2017; Updated 08 14 2017

In Fig. 4d of this Article, the schematic was incorrectly illustrated with one SIINFEKL peptide detached from a MUS/OT nanoparticle surface. The correct version of Fig. 4 appears below as Fig. 1.

## Figures and Tables

**Figure 1:**