# Supplementary material for: High-throughput quantitation of inorganic nanoparticle biodistribution at the single-cell level using mass cytometry
Source: Nat Commun. 2017 Jan 17;8:14069. doi: 10.1038/ncomms14069 (PMC5247578; doi:10.1038/ncomms14069)
Supplement: Supplementary Information — Supplementary Figures and Supplementary Table. [file ncomms14069-s1.pdf]

a

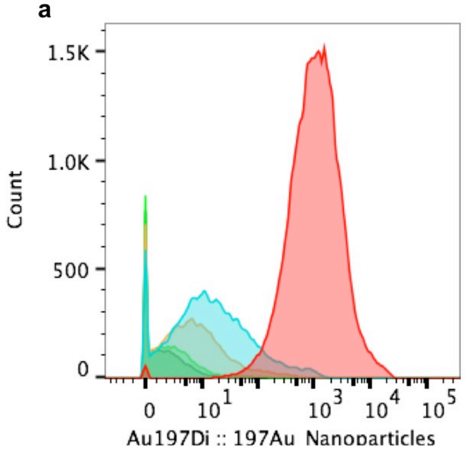

b

μg/mL	NP/ cell
0.1	1593.3 ± 19.7
0.01	17.3± 2.8
0.005	7.8 ± 2.1
0.0025	2.2 ± 0.7
0.00125	1.5± 0.6
0	0.16 ± 0.1

c

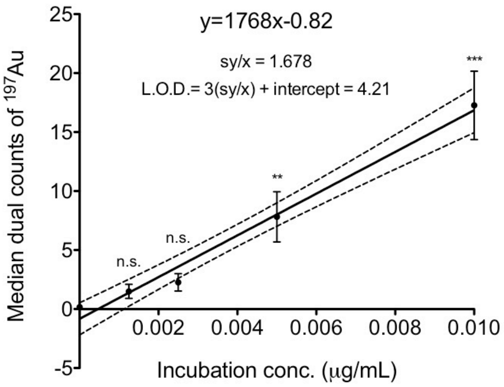

Supplementary Figure 1. CyTOF can detect as few as ~10 gold nanoparticles (~3 nm in diameter) in macrophages. RAW blue macrophages were treated with titrated concentrations of MUS/OT-BODIPY NPs: 0.1, 0.01 0.005 0.0025, or 0.00125 ug/ml for 6 hours at 37°C. (a) Representative CyTOF gold histograms in RAW cells treated with various concentrations of MUS/OT-BODIPY AuNPs. (b) Median number of NPs per cell determined from CyTOF data. (c) Regression with error shown by dashed lines (*n*=3 samples per concentration). * P < 0.05; *** P **<** 0.0001 by Bonferroni's Multiple Comparison Test compared to zero NPs per cell. Data points show mean ± s.d.

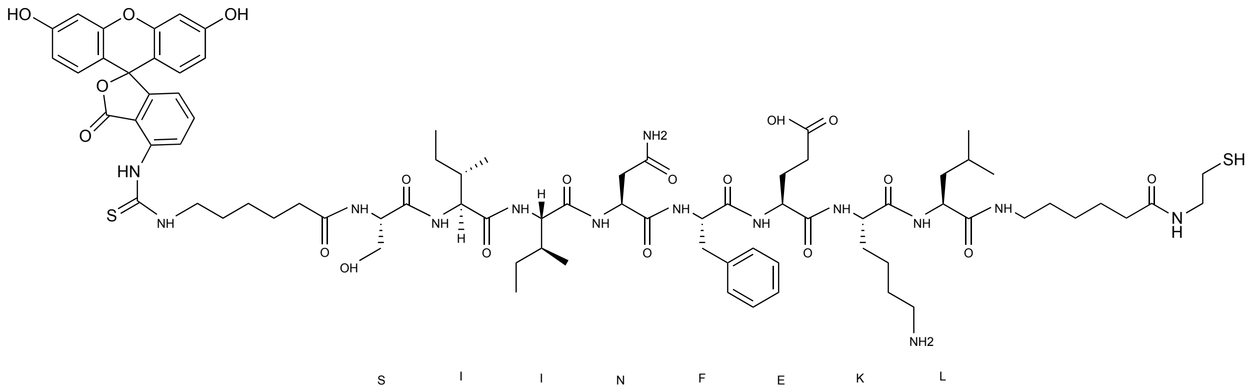

Supplementary Figure 2. Chemical structure of FITC-SIINFEKL-thiol peptide construct. This peptide was synthesized by LifeTein (Sumerset, NJ).

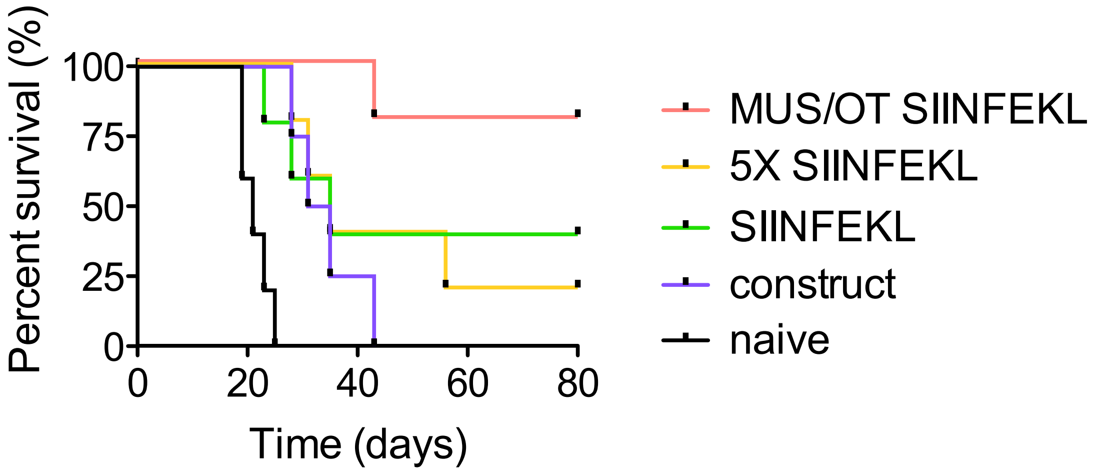

Supplementary Figure 3. Survival curve of tumor challenged mice. A quarter million of B16-OVA cells were inoculated on the flank of mice subcutaneously 150 days post vaccination.

	Core Diam. (nm)	ζ-potential (mV)
**MPSA**	**3.79±1.1**	**-32.6± 2.5**
**MUS/OT**	**2.78±0.9**	**-28.9± 2.2**
**PEG**	**2.61±1.0**	**-23.7± 0.4**

Supplementary Table 1. Characterization of nanoparticles coated with various ligands. Size distributions and zeta potentials of MPSA, MUS/OT, and PEG NPs determined from TEM and Zetasizer NanoZS, respectively.
